# Molecular identification of *Uncaria* (*Gouteng*) through DNA barcoding

**DOI:** 10.1186/s13020-015-0072-7

**Published:** 2016-02-03

**Authors:** Yin-lin Tang, Yao-sheng Wu, Rui-song Huang, Nai-xia Chao, Yong Liu, Peng Xu, Ke-zhi Li, Dan-zhao Cai, Yu Luo

**Affiliations:** Department of Biochemistry and Molecular Biology, Guangxi Medical University, 530021 Nanning, Guangxi China; Key Laboratory of Biological Molecular Medicine Research of Guangxi Higher Education, 530021 Nanning, Guangxi China; Clinical Laboratory, Maternal and Child Health Hospital of Guangxi, 530003 Nanning, Guangxi China; Guangxi Academy of Minority Nationality Medicine and Pharmacology, 530001 Nanning, Guangxi China; School of Pharmacy, Guangdong Medical College, 523808 Dongguan, Guangdong China; Liuzhou People’s Hospital, 545006 Liuzhou, Guangxi China; Affiliated Cancer Hospital of Guangxi Medical University, 530021 Nanning, Guangxi China

## Abstract

**Background:**

While DNA barcoding is an important technology for the authentication of the botanical origins of Chinese medicines, the suitable markers for DNA barcoding of the genus *Uncaria* have not been reported yet. This study aims to determine suitable markers for DNA barcoding of the genus *Uncaria* (*Gouteng*).

**Methods:**

Genomic DNA was extracted from the freshly dried leaves of *Uncaria* plants by a Bioteke’s Plant Genomic DNA Extraction Kit. Five candidate DNA barcode sites (ITS2, *rbcL*, *psbA*–*trnH*, ITS, and *matK*) were amplified by PCR with established primers. The purified PCR products were bidirectionally sequenced with appropriate amplification primers in an ABI-PRISM3730 instrument. The candidate DNA barcodes of 257 accessions of *Uncaria* in GenBank were aligned by ClustalW. Sequence assembly and consensus sequence generation were performed with CodonCode Aligner 3.7.1. The identification efficiency of the candidate DNA barcodes was evaluated with BLAST and nearest distance methods. The interspecific divergence and intraspecific variation were assessed by the Kimura 2-Parameter model. Genetic distances were computed with Molecular Evolutionary Genetics Analysis 6.0.

**Results:**

The accessions of the five candidate DNA barcodes from 11 of 12 species of *Uncaria* in China and four species from other countries were included in the analysis, while 54 of total accessions were submitted to GenBank. In a comparison of the interspecific genetic distances of the five candidate barcodes, *psbA*–*trnH* exhibited the highest interspecific divergence based on interspecific distance, theta prime, and minimum interspecific distance, followed by ITS2. The distribution of the interspecific distance of ITS2 and *psbA*–*trnH* was higher than the corresponding intraspecific distance. Additionally, *psbA*–*trnH* showed 95.9 % identification efficiency by both the BLAST and nearest distance methods regardless of species or genus level. ITS2 exhibited 92.2 % identification efficiency by the nearest distance method, but 87 % by the BLAST method.

**Conclusion:**

While *psbA*–*trnH* and ITS2 (used alone) were applicable barcodes for species authentication of *Uncaria*, *psbA*–*trnH* was a more suitable barcode for authentication of *Uncaria macrophylla*.

**Electronic supplementary material:**

The online version of this article (doi:10.1186/s13020-015-0072-7) contains supplementary material, which is available to authorized users.

## Background

*Uncaria rhynchophylla* (Miq.) Jacks is used to treat convulsion, hypertension, epilepsy, eclampsia, migraine, and cerebral diseases [1–3]. Rhynchophylline, isorhynchophylline, corynoxeine, and isocorynoxeine are the major components of *U.rhynchophylla* [4]. Oleanane and ursane-type triterpenes, (including uncarinic acids, ursolic acid, 3-hydroxyurs-12-en-27,28-dioic acid, hyperin, and catechin) were found in *Uncaria* [1, 5]. *Uncaria* comprises 34 species [6], 10 of which are found in the Guangxi Zhuang Autonomous Region. Among the 10 species of *Uncaria* in Guangxi, *U. rhynchophylla* and *Uncaria macrophylla* are the most widely and abundantly distributed [7]. Stems with hooks from several species of *Uncaria*, including *U. rhynchophylla*, *U. macrophylla*, *Uncaria hirsuta*, *Uncaria sinensis*, and *Uncaria sessilifructus*, have been used in Chinese medicine (CM) preparations, *Gouteng* in Chinese. Only the above five species plants of the genus *Uncaria* can serve as the botanical origins of *Gouteng* according to the Chinese Pharmacopeia (10th edition) [8]. Adulterants of *Gouteng* include *Uncaria laevigata*, *Uncaria lancifolia*, *Uncaria scandens*, *Uncaria rhynchophylloides*, and *Uncaria homomalla* [7, 9], due to similar organoleptic characteristics to those of *U. rhynchophylla*. But their chemical constituents and therapeutic effects are distinct from those of *U. rhynchophylla* [2, 10, 11].

DNA barcoding can accurately identify species on the basis of short standardized genes or DNA regions [12, 13], without confounding factors such as environmental influence, growth phase, and morphological diversity within species [14–16]. The mitochondrial gene encoding cytochrome c oxidase subunit 1 (*co*1) is a potential DNA barcode in most animal species as well as some fungal species. However, the *co*1 gene and other mitochondrial genes from plants have limited use in identifying plant species across a wide range of taxa, due to their low genetic variations and variable mitochondrial genomes [17]. Several DNA regions, such as ITS2, *psbA*–*trnH*, *matK*, *rbcL*, ITS, *ycf5*, and *rpoC1* [14, 18–21] have been evaluated as potential DNA barcodes in medicinal plants. Among these candidate barcoding loci, the ITS2 locus not only had the highest identification efficiency among all tested regions, but also discriminated a wide range of plant taxa [14, 22]. By contrast, ITS1 was a useful barcode for identifying *Salvia* species [23]. The *psbA–trnH* intergenic region was a suitable DNA marker for identification of flowering plants [17, 18], pteridophytes [24], *Lonicera japonica* Thunb from Caprifoliaceae [21], and aquatic plant species [25].

The authentication of the botanical origins of *Gouteng* is based on the morphological characteristics, microscopic structures, or chemical components of specimens [26]. The accuracy is often affected by environmental and subjective factors, especially for dry medicinal materials from different origins [26]. Chemical analysis methods, such as high-performance liquid chromatography (HPLC) and HPLC coupled with quadrupole time-of-flight mass spectrometry, have also been studied [27]. Multiple genetic molecular markers have been used to screen *Uncaria*, such as random amplified polymorphic DNA (RAPD) and rDNAs (including 5.8S rDNA, ITS1, and ITS2) [28].

This study aims to determine suitable markers for DNA barcoding of the genus *Uncaria*. In this study, five candidate loci (ITS2, *rbcL*, *psbA*–*trnH*, ITS, and *matK)* were tested for their potential as DNA barcodes for *Uncaria*.

## Methods

### Plant materials

Fifty-four sequences from our laboratory (all submitted to GenBank), among which 12 samples of six species of *Uncaria* (*U. rhynchophylla, U. macrophylla, U. sessilifructus, U. hirsuta, U. lancifolia* and *U. homomalla*) are used as *Gouteng* in CM markets, were collected from areas in Guangxi Province, including Rongshui, Sanjiang, Shanglin, Ningming, and Jinxi county, Nanning Sitang town, and Guangxi Medicinal Botanical Garden, in 2009 and 2010 by Professor Ruisong Huang. The plant species were identified by Shouyang Liu, Yiling Zhu, and Kejian Yan through morphological characteristics and analysis of microscopic structures [7, 10]. All of the voucher specimens (all the voucher numbers can be seen in Table 1) were deposited in the Key Laboratory of Biological Molecular Medicine Research of Guangxi Higher Education, Guangxi Medical University.

**Table 1:** *Uncaria* information used in this study

Voucher no	Species	Habitat site (county, province, country)	GenBank accession no.				
			ITS2	*rbcL*	*psbA*–*trnH*	ITS	*matK*
PS1001MT01	U. *rhynchophylla*_01	Rongshui, Guangxi, China	KM057008	KM057019	KM057031	KM057043	KM057054
PS1001MT02	*U. rhynchophylla*_02	Sanjiang, Guangxi, China	KM057009	KM057020	KM057032	KM057044	
–	*U. rhynchophylla*_03	China^a^	AJ346900			AJ346900	
PS1040MT01	*U. rhynchophylla*_04	China^a^	JF421552				
URH-1	*U. rhynchophylla*_05	China^a^	KF881222		KF881177	KF881265	
URH-2	*U. rhynchophylla*_06	China^a^	KF881223		KF881178		
PS1002MT01	*U. macrophylla*_01	Nanning, Guangxi, China	KM057010	KM057021	KM057033	KM057045	KM057055
PS1002MT02	*U. macrophylla*_02	Nanning, Guangxi, China	KM057011	KM057022	KM057034	KM057046	KM057056
PS1002MT03	*U. macrophylla*_03	Ningming, Guangxi, China	KM057012	KM057023	KM057035	KM057047	KM057057
PS1038MT03	*U. macrophylla*_04	China^a^	GQ434637	GQ436558	GQ435234		
PS1038MT04	*U. macrophylla*_05	China^a^	GQ434638	GQ436559	GQ435235		
PS1038MT01	*U. macrophylla*_06	China^a^	GQ434636				
UMA-1	*U. macrophylla*_07	China^a^	KF881209	KF881134	KF881170		
UMA-2	*U. macrophylla*_08	China^a^	KF881210	KF881135	KF881171		
UMA-3	*U. macrophylla*_09	China^a^	KF881211	KF881136	KF881172	KF881257	
UMA-4	*U. macrophylla*_10	China^a^	KF881212	KF881137	KF881173	KF881258	
UMA-5	*U. macrophylla*_11	China^a^	KF881213			KF881259	
UMA-6	*U. macrophylla*_12	China^a^	KF881214	KF881138	KF881174		
UMA-7	*U. macrophylla*_13	China^a^	KF881215				
UMA-8	*U. macrophylla*_14	China^a^	KF881216	KF881139	KF881175	KF881260	
UMA-9	*U. macrophylla*_15	China^a^				KF881261	
PS1003MT01	*U. sessilifructus*_01	Nanning, Guangxi, China	KM057013	KM057024	KM057036	KM057048	KM057058
PS1003MT02	*U. sessilifructus*_02	Shangsi, Guangxi, China			KM057037		
–	*U. sessilifructus*_03	China^a^	GU937111			GU937111	
PS1041MT02	*U. sessilifructus*_04	China^a^	GQ434640				
USE-1	*U. sessilifructus*_05	China^a^	KF881195	KF881122			
USE-2	*U. sessilifructus*_06	China^a^	KF881196	KF881123	KF881160		
USE-3	*U. sessilifructus*_07	China^a^	KF881197	KF881124	KF881161		
USE-4	*U. sessilifructus*_08	China^a^	KF881198	KF881125	KF881162		
USE-5	*U. sessilifructus*_09	China^a^	KF881199	KF881126			
USE-6	*U. sessilifructus*_10	China^a^	KF881200	KF881127			
USE-7	*U. sessilifructus*_11	China^a^	KF881201	KF881128		KF881249	
PS1004MT01	*U. hirsuta*_01	Nanning, Guangxi, China	KM057014	KM057026	KM057038	KM057049	KM057059
PS1004MT02	*U. hirsuta*_02	Nanning, Guangxi, China	KM057015	KM057027	KM057039	KM057050	KM057060
PS1004MT03	*U. hirsuta*_03	Rongshui, Guangxi, China	KM057016	KM057028	KM057040	KM057051	
–	*U. hirsuta*_04	China^a^	GU937110			GU937110	
UHI-1	*U. hirsuta*_05	China^a^	KF881235				
PS1005MT01	*U. lancifolia*_01	Jingxi, Guangxi, China	KM057017	KM057029	KM057041	KM057052	KM057061
Razafimandimbison et al. 713 (S)	*U. lancifolia*_02	Unknown^a^	KC737634	KC737740		KC737634	
ULA-1	*U. lancifolia*_03	China^a^	KF881218	KF881140	KF881176	KF881262	
ULA-2	*U. lancifolia*_04	China^a^	KF881219			KF881263	
ULA-3	*U. lancifolia*_05	China^a^	KF881220			KF881264	
ULA-4	*U. lancifolia*_06	China^a^	KF881221				
PS1006MT01	*U. homomalla*_01	Shanglin, Guangxi, China	KM057018	KM057030	KM057042	KM057053	KM057062
Munzinger 177	*U. homomalla*_02	Unkown^a^	KC737633	KC737739		KC737633	
UHO-1	*U. homomalla*_03	China^a^	KF881202	KF881129	KF881163	KF881250	
UHO-2	*U. homomalla*_04	China^a^	KF881203	KF881130	KF881164	KF881251	
UHO-3	*U. homomalla*_05	China^a^	KF881204	KF881131	KF881165	KF881252	
UHO-4	*U. homomalla*_06	China^a^	KF881205	KF881132	KF881166	KF881253	
UHO-5	*U. homomalla*_07	China^a^	KF881206		KF881167	KF881254	
UHO-6	*U. homomalla*_08	China^a^	KF881207		KF881168	KF881255	
UHO-7	*U. homomalla*_09	China^a^	KF881208	KF881133	KF881169	KF881256	
PS1039MT01	*U. sinensis*_01	China^a^	FJ980386	GQ436560	GQ435236	FJ980386	
USI-1	*U. sinensis*_02	China^a^		KF881146			
USI-2	*U. sinensis*_03	China^a^		KF881147	KF881183	KF881271	
USI-3	*U. sinensis*_04	China^a^				KF881272	
USI-4	*U. sinensis*_05	China^a^	KF881234	KF881148	KF881184	KF881273	
Razafimandimbison 304 (LBR, MO, P, TAN)	*U. africana*_01	Gabon^a^	AJ414545	AJ347006		AJ414545	
Taylor, Chanderbali, and Bourne 12075 (MO)	*U. guianensis*_01	Guyana^a^	AJ414546	AJ347007		AJ414546	
Andersson et al. 2031 (GB)	*U. tomentosa*_01	Unknown^a^	GQ852159			GQ852159	
Andersson et al. 2038 (GB)	*U. tomentosa*_02	Unknown^a^		GQ852363			
BioBot06438	*U. tomentosa*_03	Area de Conservacion Guanacaste, Rincon Rainforest, Sendero Venado, Costa Rica^a^		JQ593902			
BioBot06439	*U. tomentosa*_04	Area de Conservacion Guanacaste, Rincon Rainforest, Sendero Venado, Costa Rica^a^		JQ593903			
Razafimandimbison et al. 766 (S)	*U. lanosa*_01	Unkown^a^	KC737635	KC737741		KC737635	
UYU-1	*U. yunnanensis*_01	China^a^	KF881243	KF881156	KF881191	KF881281	
UYU-2	*U. yunnanensis*_02	China^a^	KF881244				
UYU-3	*U. yunnanensis*_03	China^a^	KF881245	KF881157		KF881282	
UYU-4	*U. yunnanensis*_04	China^a^	KF881246	KF881158	KF881193	KF881283	
UYU-5	*U. yunnanensis*_05	China^a^	KF881247		KF881194		
UYU-6	*U. yunnanensis*_06	China^a^	KF881248	KF881159		KF881284	
WP2E0309	*U. appendiculata*_01	Papua New Guinea^a^		JF738785			
WP1D0176	*U. appendiculata*_02	Papua New Guinea^a^		JF738676			
WP5E1207	*U. appendiculata*_03	Papua New Guinea^a^		JF739007			
Razafimandimbison et al. 768 (S)	*U. scandens*_01	Unknown^a^	KC737636	KC737742		KC737636	
USC-1	*U. scandens*_02	China^a^	KF881236	KF881149	KF881185	KF881274	
USC-2	*U. scandens*_03	China^a^	KF881237	KF881150	KF881186	KF881275	
USC-3	*U. scandens*_04	China^a^	KF881238	KF881151	KF881187	KF881276	
USC-4	*U. scandens*_05	China^a^	KF881239	KF881152	KF881188	KF881277	
USC-5	*U. scandens*_06	China^a^	KF881240	KF881153		KF881278	
USC-6	*U. scandens*_07	China^a^	KF881241	KF881154	KF881189	KF881279	
USC-7	*U. scandens*_08	China^a^	KF881242	KF881155	KF881190	KF881280	
HITBC:Liana Mengsong 107_7_4	*U. laevigata*_01	Mengsong, Yunnan, China^a^		KF181471			HG004898
ULAE-1	*U. laevigata*_02	China^a^	KF881224	KF881142	KF881179	KF881266	
ULAE-2	*U. laevigata*_03	China^a^	KF881225			KF881267	
ULAE-3	*U. laevigata*_04	China^a^	KF881226	KF881143		KF881268	
ULAE-4	*U. laevigata*_05	China^a^	KF881227	KF881144	KF881180	KF881269	
ULAE-5	*U. laevigata*_06	China^a^	KF881228		KF881181		
ULAE-6	*U. laevigata*_07	China^a^	KF881229			KF881270	
ULAE-7	*U. laevigata*_08	China^a^	KF881230		KF881182		
Total no. of sequences		257	77	63	49	58	10

^a^From GenBank

In total, 257 accessions related to the five candidate DNA barcoding sites (ITS2, *rbcL*, *psbA*–*trnH*, ITS, and *matK*) from 89 samples belonging to 15 species of *Uncaria* were analyzed in this study. All accession data were downloaded from GenBank, except for the above 54 sequences, which were amplified and sequenced in our laboratory. All datasets of *Uncaria* species used in the study contained more than two samples, except for *Uncaria africana*, *Uncaria guianensis*, and *Uncaria lanosa*. Some accessions in which the sequences contained undetermined bases or were from sp. species (taxa of species unclear or unnamed) were not selected. In this study, the correctness of the accessions downloaded from GenBank was tested through blasting against those of congener plants. Only the sequences with both a similarity ratio and query cover ratio higher than 90 % in the same species were suitable for selection. However, some accessions containing inversion sequences were collected in this dataset because they could influence the sequence divergence and supply some important genetic characters [29]. The total data and sample information used in this study are shown in Table 1.

### DNA extraction, PCR amplification, and sequencing

In this study, genomic DNA was extracted from the freshly dried leaves of *Uncaria* plants by the improved protocol of a new rapid Plant Genomic DNA Extraction Kit (centrifugal column type, DP3112; Bioteke Corporation, Beijing, China). The *Uncaria* leaves were ground in liquid nitrogen, and the cell nuclear separation solution (3 ml for 0.5 g sample) was immediately added to the samples to remove impurities from the cytoplasm before the cell nuclei were lysed [30]. PCR amplification of the five candidate DNA barcode sites was performed in a Tprofessional Gradient 96 Type (Biometra, Göttingen, Germany) with approximately 30 ng of genomic DNA as a template in a 25-µL reaction mixture. Each reaction contained 1 × PCR buffer (2.0 mM MgCl_2_, 0.2 mM each dNTP, 0.1 µM each primer; synthesized by Sangon Biotech, Co., Ltd., Shanghai, China), and 1.0 U Taq DNA polymerase (TaKaRa Biotechnology Co., Ltd., Dalian, China). The primers and reaction conditions used were the same as those used by Chen et al. [14]. The PCR products were electrophoresed in a 1.5 % agarose gel in 1 × TAE buffer, then purified with a TIANGel Midi Purification Kit (Tiangen Biotech Co. Ltd, Beijing, China). The purified PCR products were bidirectionally sequenced with appropriate amplification primers (Additional file 1) in an ABI-PRISM3730 instrument (Thermo Fisher Scientific, MA, USA) by Sangon Biotech, Co., Ltd., Shanghai, China.

### Sequence alignment and data analysis

Sequence assembly and consensus sequence generation were performed by CodonCode Aligner 3.7.1 (CodonCode Co., MA, USA) by trimming the low quality sequence and primer areas. The *matK* and *rbcL* regions were delimited by alignment with known sequences in databases by CodonCode Aligner. After removal of the *psbA* and *trnH* genes at the ends of *psbA*–*trnH*, the boundary of the *psbA*–*trnH* intergenic spacer was determined according to the annotations of similar sequences in GenBank. The five candidate DNA barcodes were aligned by ClustalW (EMBL-EBI, Heidelberg, German). Kimura 2-Parameter (K2P) genetic distances were computed with Molecular Evolutionary Genetics Analysis 6.0 (The Biodesign Institute, AZ, USA) [31]. All interspecific and intraspecific distances, including theta prime, minimum interspecific distance, theta, and coalescent depth for all accessions of each locus, were calculated and compared to evaluate the interspecific divergence and intraspecific variation by the K2P model. Meanwhile, statistical analysis of the distribution divergency of the genetic distance between different sequences was performed through the Wilcoxon signed-rank test to assess the barcoding gap for different candidate loci with SPSS software (SPSS 16.0: International Business Machines Corporation Statistical Product and Service Solutions, Armonk, New York, USA), which the test statistical W+ and W− were calculated for two side test, as described previously [14, 22]. The BLAST1 and nearest distance methods were used to evaluate the species identification efficiency [32, 33].

## Results

### PCR amplification and base composition of the five loci of *Uncaria*

The sequence length and GC content of the five candidate loci (ITS2, *rbc*L, *psbA*–*trnH*, ITS, and *matK*) were obtained from the CodonCode Aligner and Clustal W alignment results (Table 2). The GC content of *psbA*–*trnH* was the lowest, while that of ITS2 was the highest. The variability of the length range of the *psbA*–*trnH* intergenic spacer was greater than that of the other candidates. The *psbA*–*trnH* region of *U. macrophylla* was more divergent than that of the other *Uncaria* plants.

**Table 2:** Analysis of the five candidate barcode loci of *Uncaria*

Items	ITS2	*rbcL*	*psbA*–*trnH*	ITS	*matK*
Species numbers	14	15	10	14	7
Accession no.	77	63	49	58	10
Length range (average) (bp)	210–221 (220)	512–656 (608)	235–315 (287)	607–621 (616)	757–814 (808)
Average of GC content (%)	66.3	43.0	24.8	62.8	33.1
No. of variable sites in all taxa	41	16	173	86	13
No. of indels in all taxa	2	0	39	14	0
BLAST method (identification efficiency [%])	87.0	42.9	95.9	91.4	80
Nearest distance method (identification efficiency [%])	92.2	76.2	95.9	84.5	80

### Genetic interspecific divergence and intraspecific variation

**Fig. 1:**
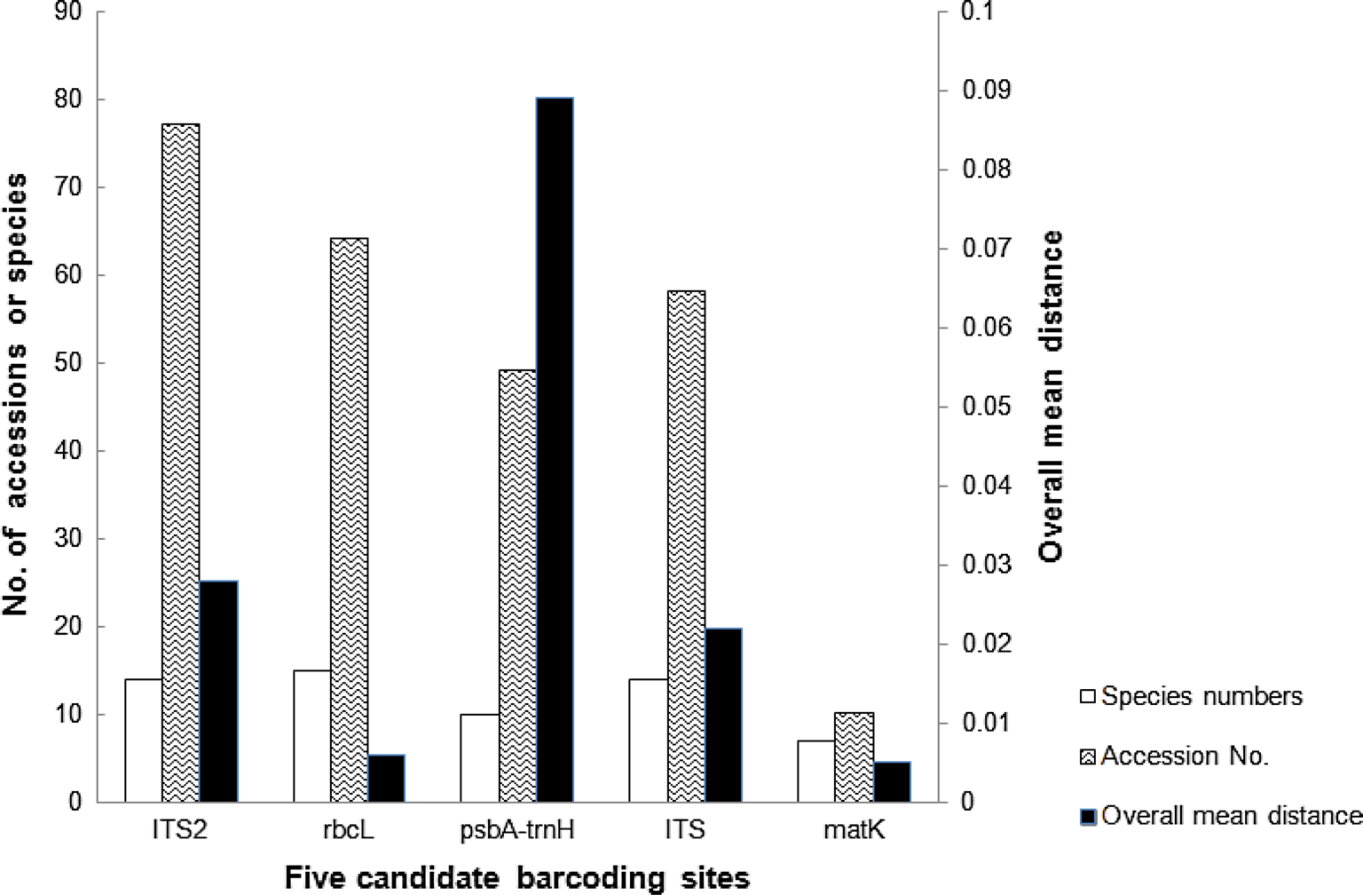
Distribution of overall mean distance for all sequence pairs among five loci. The number at right y axis is the estimates of average evolutionary divergence over all sequence pairs for each locus, which is the base substitutions per site from averaging over all sequence pairs. Analyses were conducted by the maximum composite likelihood method in MEGA6 [31]

Six parameters (Table 3) represented the genetic divergences of species in *Uncaria*. In a comparison of the intraspecific distances of the five candidate barcodes among *Uncaria* species, the intraspecific distance of *psbA*–*trnH* was higher than that of the other loci at the species level. Meanwhile, the interspecific genetic distance of the *psbA*–*trnH* intergenic spacer exhibited the highest divergence according to the interspecific distance, theta prime, and minimum interspecific distance. The interspecific distance of ITS2 was the second highest after *psbA*–*trnH*. All interspecific divergences of ITS2, *psbA*–*trnH*, and ITS were greatly higher than the corresponding intraspecific divergences. Furthermore, the overall mean distance of *psbA*–*trnH* was the highest among the five loci (Fig. 1).

**Table 3:** Calculation of interspecific and intraspecific divergences for *Uncaria*

Parameters	ITS2	*rbcL*	*psbA*–*trnH*	ITS	*matK*
Intraspecific divergence theta	0.0044 ± 0.0063	0.0010 ± 0.0013	0.0674 ± 0.0508	0.0080 ± 0.0089	0.0010 ± 0.0003
Coalescent depth	0.0171 ± 0.0292	0.0022 ± 0.0025	0.1060 ± 0.0705	0.0153 ± 0.0151	0.0012 ± 0.0000
All intraspecific distance	0.0059 ± 0.0128	0.0010 ± 0.0021	0.0480 ± 0.0401	0.0047 ± 0.0079	0.0009 ± 0.0006
Theta prime	0.0340 ± 0.0089	0.0040 ± 0.0021	0.0986 ± 0.0299	0.0253 ± 0.0050	0.0060 ± 0.0024
Minimum interspecific distance	0.0151 ± 0.0141	0.0009 ± 0.0017	0.0192 ± 0.0232	0.0104 ± 0.0092	0.0030 ± 0.0028
All interspecific distance	0.0348 ± 0.0166	0.0042 ± 0.0033	0.1068 ± 0.0468	0.0239 ± 0.0102	0.0057 ± 0.0027

The *psbA*–*trnH* intergenic spacer had the highest interspecific divergence among all the loci based on the Wilcoxon signed-rank test. The second highest interspecific divergence was shown by ITS2. The scale of the interspecific divergence of *psbA*–*trnH* was higher than ITS2, ITS, *matK* and *rbcL*, respectively (all *P* < 0.001), that of ITS2 was higher than ITS, *matK* and *rbcL*, respectively (all *P* < 0.001, Table 4). Furthermore, the intraspecific divergences between ITS and *matK*, *rbcL* and *matK*, ITS2 and *matK*, *psbA*–*trnH* and *matK*, and ITS and *rbcL* did not exhibit any significant differences (*P* > 0.05, Table 5).

**Table 4:** Wilcoxon signed-rank test for interspecific divergences

W+	W−	Inter relative rank	n	*P* value	Result
ITS2	*rbcL*	W+ = 1.00, W− = 639.50	1282	2.25 × 10^−210^	ITS2 > *rbcL*
ITS2	*psbA*–*trnH*	W+ = 506.72, W− = 98.66	957	1.42 × 10^−149^	ITS2 < *psbA*–*trnH*
ITS2	ITS	W+ = 365.08, W− = 744.61	1358	8.42 × 10^−143^	ITS2 > ITS
ITS2	*matK*	W+ = 0.00, W− = 16.50	32	7.93 × 10^−7^	ITS2 > *matK*
*rbcL*	*psbA*–*trnH*	W+ = 360.00, W− = 0.00	719	2.27 × 10^−119^	*rbcL* < *psbA*–*trnH*
*rbcL*	ITS	W+ = 442.81, W− = 20.38	862	8.05 × 10^−141^	*rbcL* < ITS
*rbcL*	*matK*	W+ = 22.63, W− = 17.86	41	0.0193	*rbcL* < *matK*
*psbA*-*trnH*	ITS	W+ = 27.27, W− = 287.47	560	1.80 × 10^−92^	*psbA*-*trnH* > ITS
*psbA*-*trnH*	*matK*	W+ = 0.00, W− = 16.50	32	7.93 × 10^−7^	*psbA*-*trnH* > *matK*
ITS	*matK*	W+ = 0.00, W− = 16.50	32	7.93 × 10^−7^	ITS > *matK*

**Table 5:** Wilcoxon signed-rank test for intraspecific divergences

W+	W−	Intra relative rank	n	*P* value	Result
ITS2	*rbcL*	W+ = 23.12, W− = 45.44	149	7.54 × 10^−6^	ITS2 > *rbcL*
ITS2	*psbA*–*trnH*	W+ = 60.70, W− = 11.00	124	1.90 × 10^−20^	ITS2 < *psbA*–*trnH*
ITS2	ITS	W+ = 49.59, W− = 37.93	127	0.0166	ITS2 > ITS
ITS2	matK	W+ = 2.00, W− = 0.00	4	0.1025	ITS2 = *matK*
*rbcL*	*psbA*–*trnH*	W+ = 46.00, W− = 0.00	101	1.19 × 10^−16^	*rbcL* < *psbA*–*trnH*
*rbcL*	ITS	W+ = 29.17, W− = 26.60	84	0.3788	*rbcL* = ITS
*rbcL*	*matK*	W+ = 2.00, W− = 0.00	4	0.1025	*rbcL* = *matK*
*psbA*–*trnH*	ITS	W+ = 10.50, W− = 34.22	70	4.23 × 10^−12^	*psbA*–*trnH* > ITS
*psbA*–*trnH*	*matK*	W+ = 1.00, W− = 2.50	4	0.2763	*psbA*–*trnH* = *matK*
ITS	*matK*	W+ = 2.00, W− = 0.00	4	0.1025	ITS = *matK*

### Analysis of barcoding gaps

**Fig. 2:**
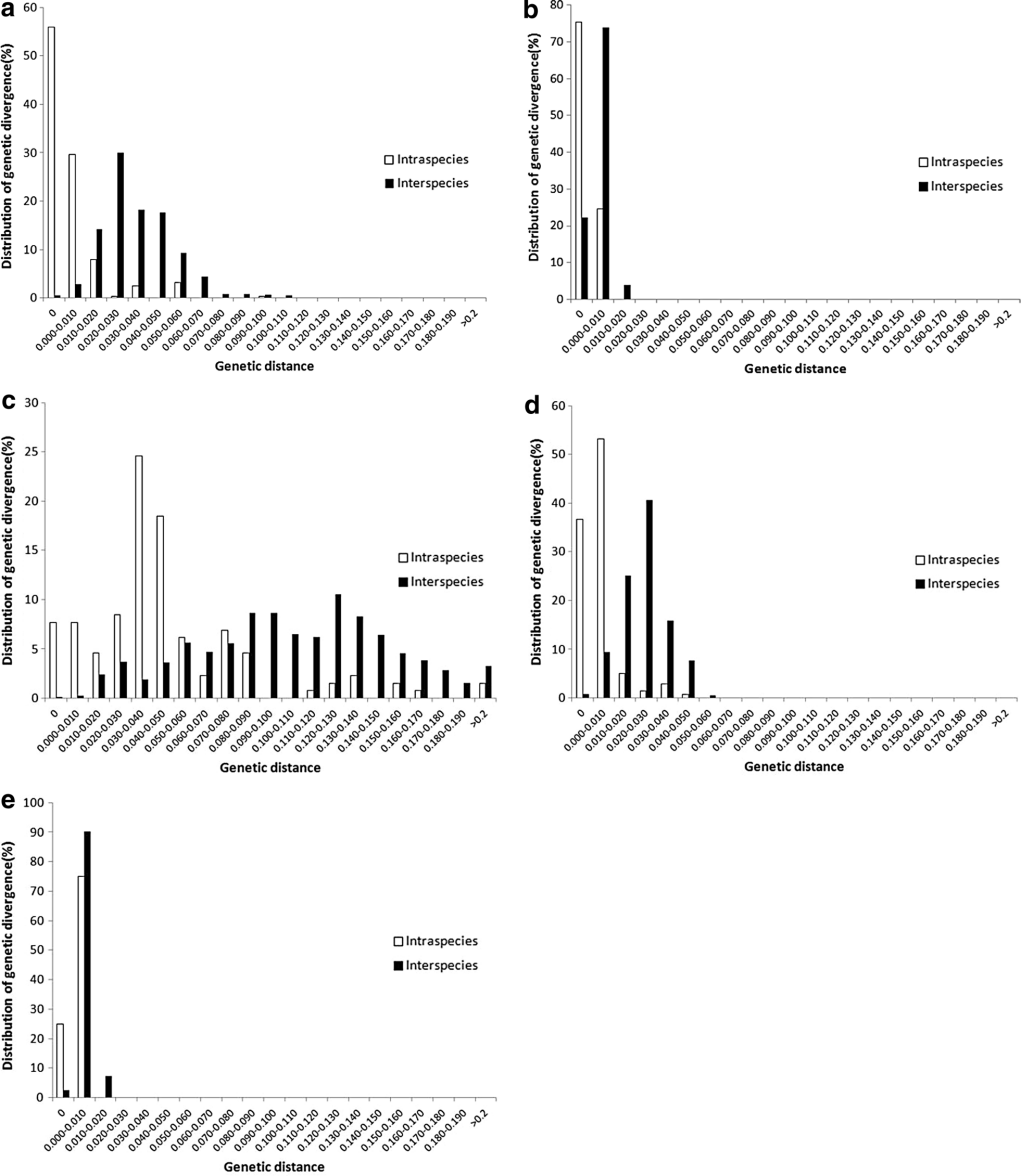
Distribution of divergence between interspecific and intraspecific genetic distance for five candidate barcoding loci among 257 accessions. **a** ITS2; **b** *rbcL*; **c** *psbA*–*trnH*; **d** ITS;** e**, *matK*.* x axis*, genetic distance;* y axis*, distribution of genetic divergence

As a barcode for identifying botanical species, the divergence between species should be higher than the variation within species [34]. Although the histogram of the K2P genetic distance analysis revealed a partial overlap “barcoding gap” between the intraspecific and interspecific divergence of ITS2 or *psbA*–*trnH* (Fig. 2), the intraspecific variation of *psbA*–*trnH* and ITS2 was considerably lower than the distribution of their interspecific divergence. The genetic divergence distribution of ITS was similar to that of ITS2. No clear “barcoding gap” corresponding to the *rbcL* or *matK* loci was observed, wherein the genetic distance distribution of more than 90 % of accessions was less than 0.020. However, the distribution of the interspecific divergence of ITS2 and *psbA*–*trnH* provided a better resolution than that of *rbcL* and *matK*.

### Identification efficiency and characteristics of Clustal W alignment

**Fig. 3:**
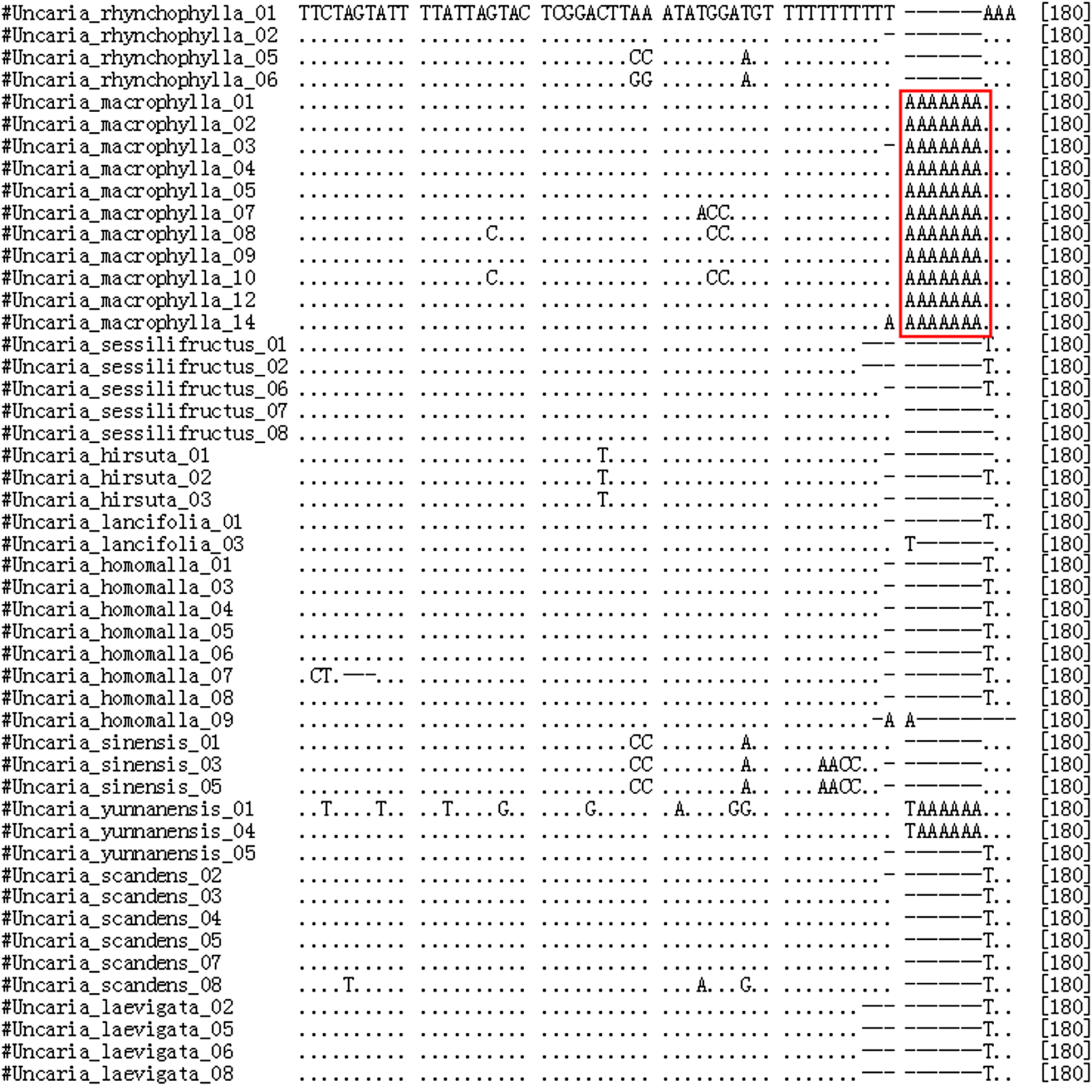
ClustalW results of *psbA*–*trnH* of *Uncaria* plants. Identical positions are shown as* dot*; indels as* dash*; the *red box site* show a seven A repeat inserted at 171–177 bp, the differences of *U. macrophylla* from *U. rhychophylla* and other *Uncaria* species

**Fig. 4:**
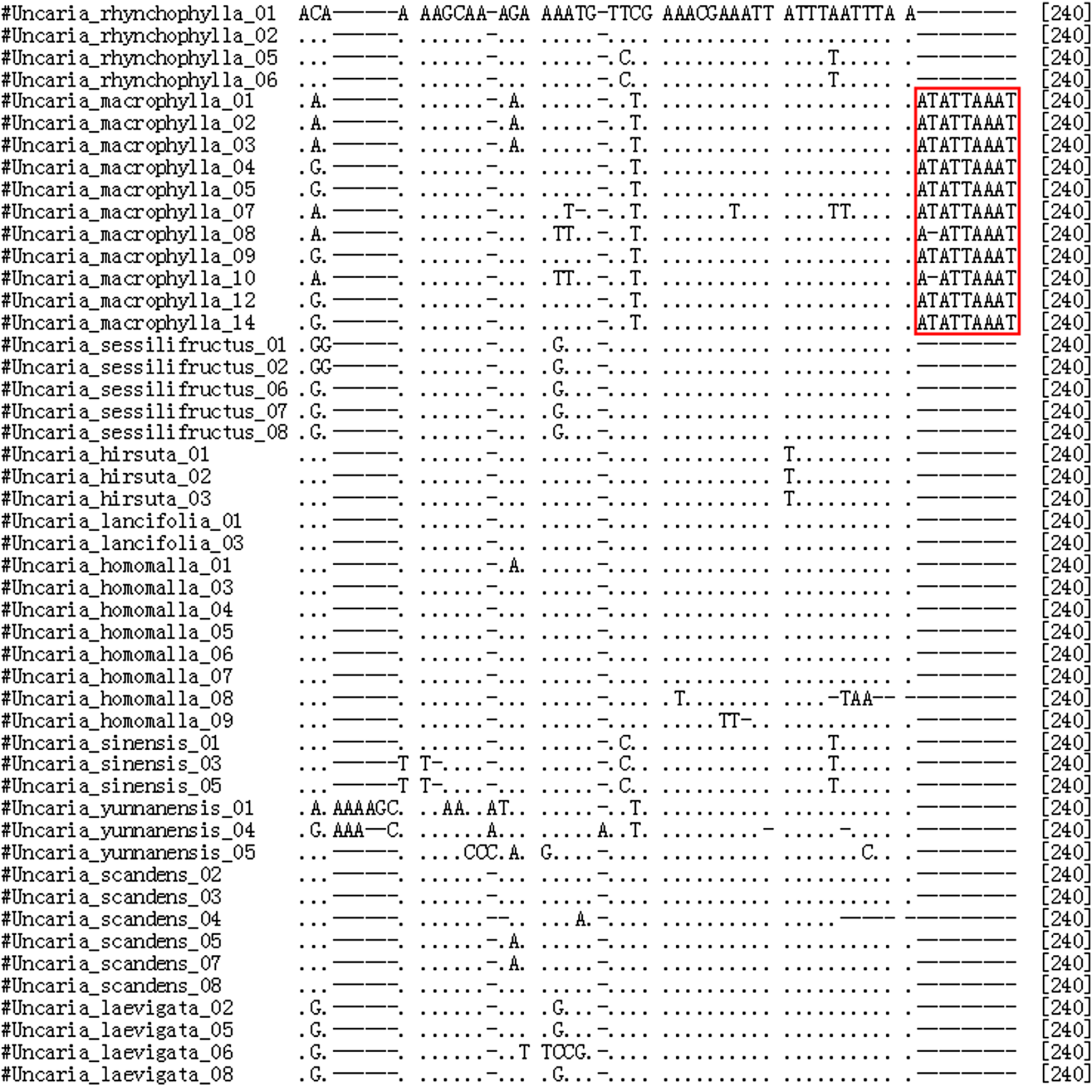
ClustalW results of *psbA*–*trnH* of *Uncaria* plants. Identical positions are shown as* dot*; indels as* dash*; the *red box site* show a cis-repeats of ATTAAA insertion at 234–239 bp, the differences of *U. macrophylla* from *U. rhychophylla* and other *Uncaria* species

**Fig. 5:**
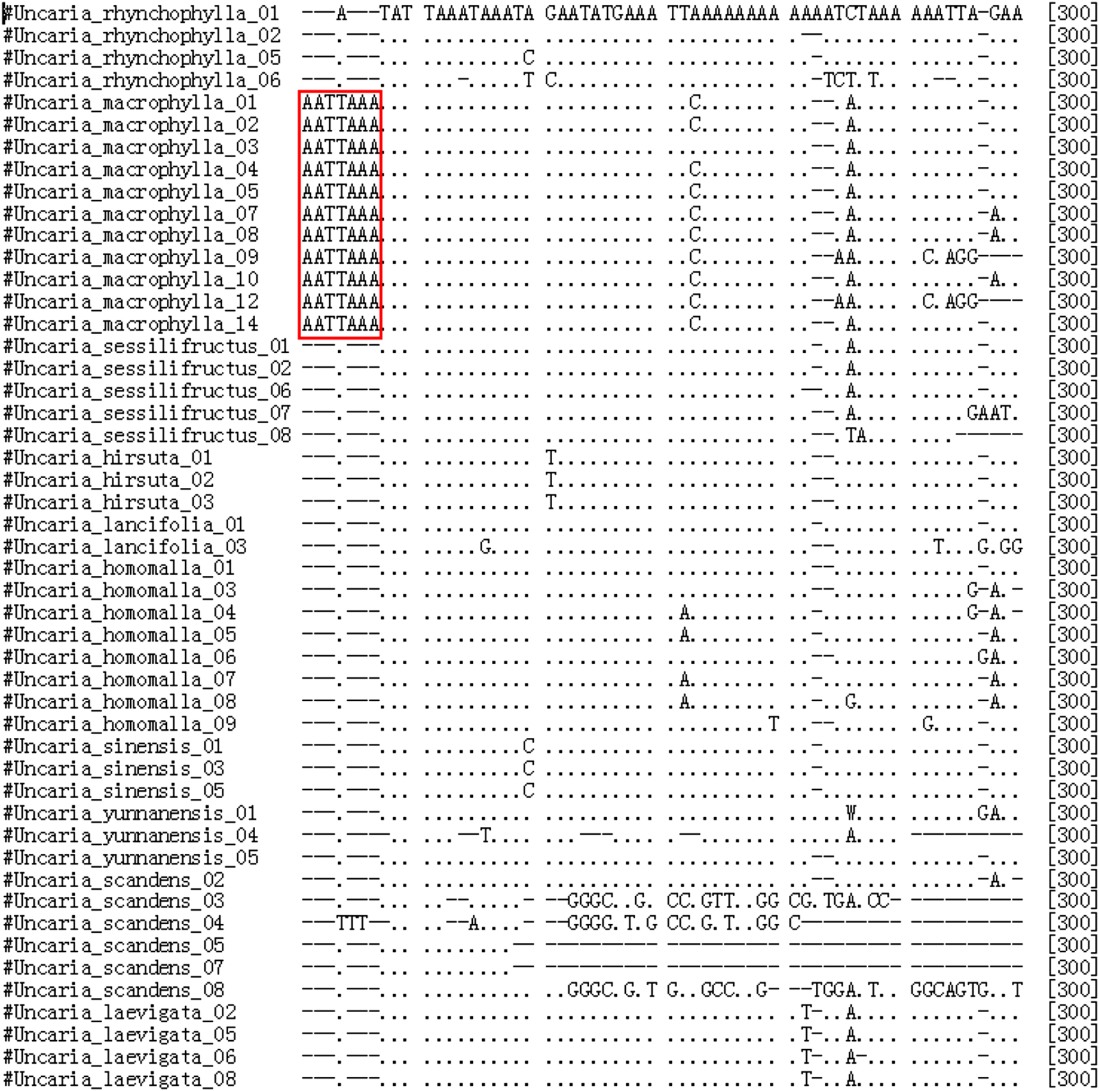
ClustalW results of *psbA*–*trnH* of *Uncaria* plants. Identical positions are shown as* dot*; indels as* dash*; the *red box site* show a cis-repeats of ATTAAA insertion at 241-247 bp, the differences of *U. macrophylla* from *U. rhychophylla* and other *Uncaria* species

**Fig. 6:**
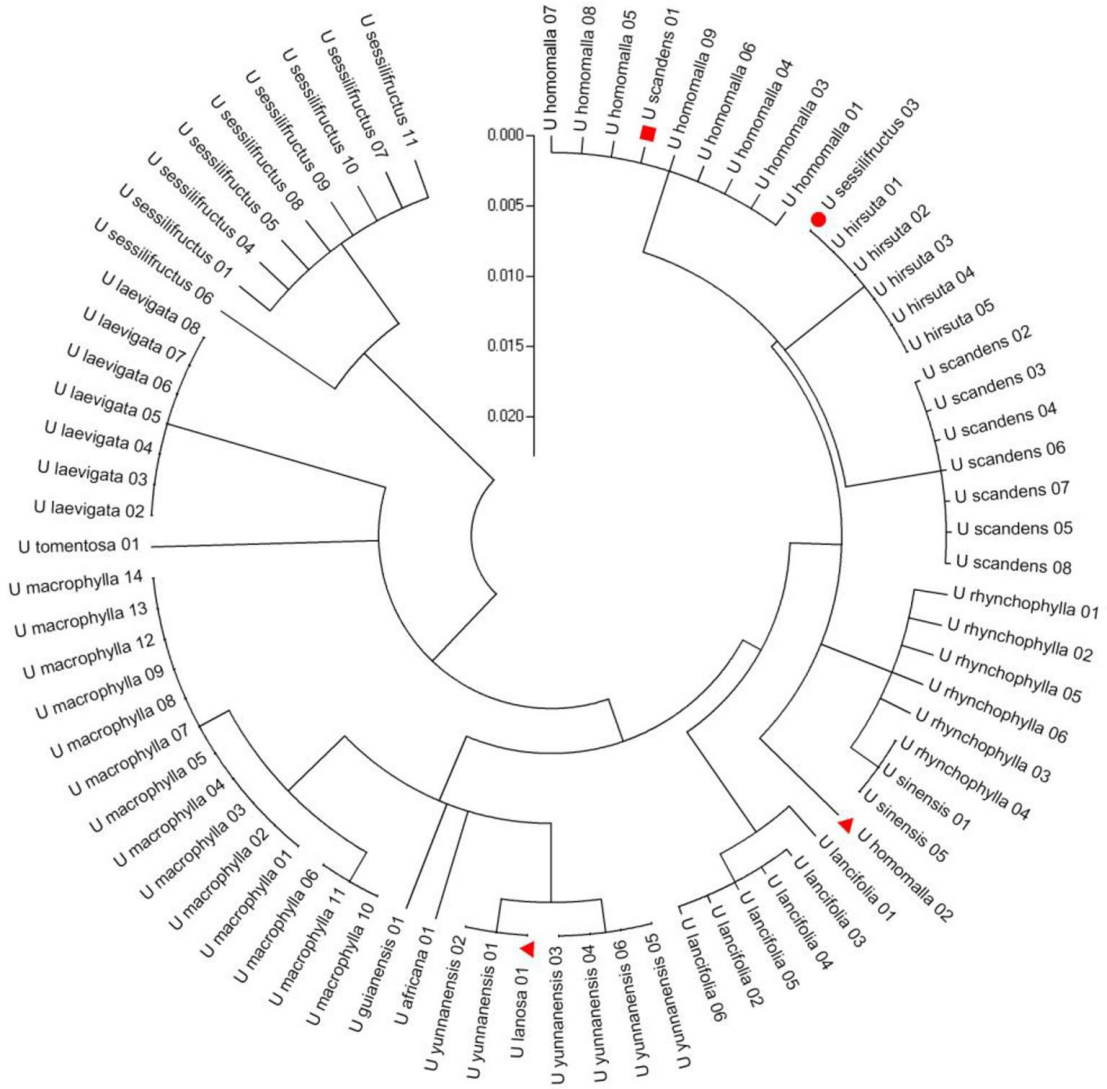
Phylogeny tree of *Uncaria* ITS2. The evolutionary history was inferred using the neighbor-joining method, the evolutionary distances were computed using the maximum composite likelihood model. Only four accessions labeled by *triangular*, *square* or *circular* symbol were incorrectly taxonomic category

The BLAST and nearest distance methods were employed to test the applicability of the five loci for species identification of *Uncaria*. *psbA*–*trnH* presented 95.9 % identification efficiency with both the BLAST and nearest distance methods at the species or genus level. ITS2 exhibited 92.2 % identification efficiency by the nearest distance method, but 87 % by the BLAST method, whereas *rbcL* showed only 76.2 % by the nearest distance method and 42.9 % by the BLAST method (Table 2). Meanwhile, *psbA*–*trnH* of *U. macrophylla* exhibited more obvious characteristics than *U. rhynchophylla* and the other species tested (Figs. 3, 4, 5). Two insertion fragments existed in the *psbA*–*trnH* sequence of *U. macrophylla*, including a serial seven A fragment at 171–177 bp, and another double repeat “ATTAAA” at 234–247 bp. The *psbA*–*trnH* intergenic spacer can be used as a barcode for the identification of *Uncaria* plants. The phylogeny of *Uncaria* ITS2 (computed model: Maximum Composite Likelihood) [31] showed that only four accessions (4/77 accessions) were in the incorrect taxonomic category (Fig. 6), which was less than the other loci tested. Thus, ITS2 could be another suitable DNA barcode for *Uncaria*.

## Discussion

### Significance of authentication of *Uncaria* by DNA barcoding

*Gouteng* is commonly exploited as the major ingredient herb of CM prescriptions for hypertension or migraine treatment [2, 35]. The amount of stems with hooks of *U. rhynchophylla* (*Gouteng*) required in traditional clinic and pharmaceutical production, has been increased; while the natural growth of *U. rhynchophylla, U. hirsuta* and *U. macrophylla* which could serve as the botanical origins of *Gouteng* was limited with the rising of collection. Some other species of the genus *Uncaria* are often collected to adulterate *Guoteng*, such as *U. laevigata*, *U. lancifolia*, *U. scandens* [7]. Therefore, the correct genotypic identification of *Uncaria* plant material is essential in order to protect public health and for industrial production.

Although some methods have been developed to distinguish *Uncaria* plants based on morphotype, microcharacter, or physical and chemical reactions [8, 9], these are dependent on taxonomy experts. Currently, the genetic molecular markers for the genus *Uncaria* were related to RAPD, rDNA, and ITS, while DNA barcoding assays have not yet been reported. This study included 11 of 12 species of *Uncaria* in China, with *U. rhynchophylloides* missing in the screen for suitable DNA barcodes for *Uncaria*.

In the present study, *psbA*–*trnH* presented 95.9 % identification efficiency for *Uncaria* accessions tested with both BLAST and nearest distance methods at the species or genus level. ITS2 also exhibited high identification efficiency at 92.2 or 87 % with the nearest distance or BLAST method, respectively.

### Quality and amplification efficiency of DNA from *Uncaria*

The DNA of *Uncaria* was not extracted efficiently, due to the large amounts of polysaccharides, polyphenols, and alkaloids present in the samples. A cell nuclear separation solution was used to remove the impurities from genomic DNA [30]. The quality of the DNA extracted from the *Uncaria* plants satisfied the requirements for PCR amplification and sequencing. The efficiency of both PCR amplification and sequencing for *psbA*–*trnH* was the highest among the five candidate loci. Specifically, PCR amplification showed 96.7 % efficiency, while sequencing showed 100 % efficiency. Because the average GC content of ITS2 was 66.3 %, which was higher than that of the other loci, the resulting DNA extract was slightly difficult to amplify.

### Selection of candidate DNA barcodes

In this study, the length of *psbA*–*trnH* of *Uncaria* ranged from 235 to 315 bp (mean 287 bp), which was longer than that of ITS2, but shorter than that of *rbcL*, ITS, and *matK*. Additionally, *psbA*–*trnH* of *Uncaria* exhibited the highest interspecific divergence among the five loci tested, based on the results of six parameters of the K2P model or Wilcoxon signed-rank test of interspecific divergence. The interspecies divergence of *psbA*–*trnH* was higher than the relevant intraspecies variation. Furthermore, *psbA*–*trnH* of *U. macrophylla* was significantly distinct from that of *U. rhynchophylla* and the other species because of two insertion fragments: one was a seven A repeat inserted at 171–177 bp and the other was two cis-repeats of ATTAAA at 233–247 bp (Figs. 3, 4, 5). Although one TAAAAAA repeat was observed at 171–177 bp in *psbA*–*trnH* from *Uncariayunnanensis*, no double cis-repeats of ATTAAA were observed at 233–247 bp. Meanwhile, one inversion sequence of length 73–74 bp with identity ratios of more than 98 % in *psbA*–*trnH* of *Uncaria* was found in this study (Additional file 2). The intragenic variation of the genus *Uncaria* was large because of this inversion phenomenon existing in *psbA*–*trnH*. This situation was also observed in *psbA*–*trnH* of *Aconitum* L. [29]. The characteristics of the insertion sequences in *psbA*–*trnH* could effectively authenticate *Uncaria* species.

ITS2 was another suitable locus for distinguishing different species of *Uncaria*. The length range of ITS2 was 210–221 bp (mean 219.9 bp), which was the shortest among the five loci. Consequently, 95.8 % efficiency could be reached by PCR amplification. In a comparison of the interspecific genetic distances of the five candidate barcodes among *Uncaria* species, the mean interspecific distance of ITS2 was higher than its mean intraspecific divergence, and the values were second only to those of *psbA*–*trnH* (Table 3). Based on the phylogenetic analysis of ITS2 by the neighbor-joining method and the evolutionary distances computed by the Maximum Composite Likelihood model, more than 93 % of *Uncaria* at the species level in this study were divided into monophyla as recognized species. Among 77 accessions of ITS2, comprising 14 species of *Uncaria*, only four accessions were in an incorrect taxonomic category, according to the construction of a phylogenetic tree for ITS2 (Fig. 6). *Uncaria* manifested complex morphological features and genetic backgrounds, and even some specimens with obvious differences in appearance possessed similar ITS sequences [28]. This could explain the existence of some accessions that appeared in different monophyla from their original morphological taxa. Some species submitted to GenBank may have been wrongly categorized. Sequences with lengths of less than 100 bp, those with ambiguous bases containing more than one “N”, or those belonging to unnamed species (such as those with spp. and aff. in the species name) were excluded [20] from this study to guarantee the reliability of the selected sequences.

A better “barcoding gap” was observed between the interspecific divergence and intraspecific variation of ITS2 compared with the other loci. ITS, which contained three fragments (ITS1, 5.8S rDNA, ITS2), exhibited a similar identification efficiency to that of ITS2. Both *rbcL* and *matK* were unsuitable genetic loci for authentication of the botanical origins of *Gouteng*, because of the absence of a clear barcoding gap between the interspecific divergence and intraspecific variation by the K2P model. The overall mean distance of *rbcL* was only 0.002 and that for *matK* was 0.005, as computed by the Maximum Composite Likelihood model (Fig. 1). Moreover, we found that the combination of *psbA*–*trnH* with ITS2 would provide a better result for the authentication of *Uncaria* plants, and could even distinguish between incorrect and correct taxa or identify some cryptic species. Currently, a preliminary system for DNA barcoding of herbal materials has been established based on a two-locus combination of ITS2 and *psbA*–*trnH* barcodes [36]. Recently, ITS2 was successfully exploited in a survey involving commercial *Rhodiola* products, including decoction pieces [37].

*psbA*–*trnH* and ITS2 also exhibited high authentication power for different species of *Uncaria*. Both *psbA*–*trnH* and ITS2 revealed the distinct divergence of *U. macrophylla* from *U. rhynchophylla* and the other species at the species level.

## Conclusion

While *psbA*–*trnH* and ITS2 (used alone) were applicable barcodes for species authentication of *Uncaria*, *psbA*–*trnH* was a more suitable barcode for authentication of *U. macrophylla*.

## Additional files

10.1186/s13020-015-0072-7 The univeral primers for candidate barcodes PCR amplication and sequening in the study.
10.1186/s13020-015-0072-7 The accessions containing inversion sequence in *psbA-trnH of Uncaria*.

## Abbreviations

ITS: internal transcribed spacer
*psbA*–*trnH*: gene spacer between *psbA* and *trnH* in chloroplast DNA
K2P: Kimura 2-Parameter
CM: Chinese medicine
HPLC: high-performance liquid chromatography
RAPD: random amplified polymorphic DNA
