# Supplementary material for: Molecular identification of Uncaria (Gouteng) through DNA barcoding
Source: Chin Med. 2016 Feb 3;11:3. doi: 10.1186/s13020-015-0072-7 (PMC4739391; doi:10.1186/s13020-015-0072-7)
Supplement: Supplementary file 1 — 10.1186/s13020-015-0072-7 The univeral primers for candidate barcodes PCR amplication and sequening in the study. [file 13020_2015_72_MOESM1_ESM.docx]

List of universal primers for candidate barcodes sequencing

| **Marker** | | **Name of primers** | **Primer sequences 5′-3′** |
| --- | --- | --- | --- |
| **ITS** | | 5a fwd | CCTTATCATTTAGAGGAAGGAG |
|  | | 4 rev | TCCTCCGCTTATTGATATGC |
| **ITS2** | | S2F | ATGCGATACTTGGTGTGAAT |
|  | | S3R | GACGCTTCTCCAGACTACAAT |
| ***rbcL*** | | 1f | ATGTCACCACAAACAGAAAC |
|  | | 724r | TCGCATGTACCTGCAGTAGC |
| ***psbA-trnH*** | | fwd PA | GTTATGCATGAACGTAATGCTC |
|  | | rev TH | CGCGCATGGTGGATTCACAATCC |
| ***matK*** | | 390F | CGATCTATTCATTCAATATTTC |
|  | 1326R | | TCTAGCACACGAAAGTCGAAGT |
