# Supplementary material for: Molecular identification of Uncaria (Gouteng) through DNA barcoding
Source: Chin Med. 2016 Feb 3;11:3. doi: 10.1186/s13020-015-0072-7 (PMC4739391; doi:10.1186/s13020-015-0072-7)

The accessions containing inversion sequence:

| voucher No | species | *psbA-trnH* | Inversion |
| --- | --- | --- | --- |
| PS1001MT01 | U. *rhynchophylla*_01 | KM057031 | Yes |
| PS1001MT02 | *U. rhynchophylla*_02 | KM057032 | Yes |
| URH-1 | *U. rhynchophylla*_05 | KF881177 | Yes |
| URH-2 | *U. rhynchophylla*_06 | KF881178 | Yes |
| PS1002MT01 | *U. macrophylla*_01 | KM057033 | Yes |
| PS1002MT02 | *U. macrophylla*_02 | KM057034 | Yes |
| PS1002MT03 | *U. macrophylla*_03 | KM057035 | Yes |
| PS1038MT03 | *U. macrophylla*_04 | GQ435234 | Yes |
| PS1038MT04 | *U. macrophylla*_05 | GQ435235 | Yes |
| UMA-1 | *U. macrophylla*_07 | KF881170 | Yes |
| UMA-2 | *U. macrophylla*_08 | KF881171 | Yes |
| UMA-3 | *U. macrophylla*_09 | KF881172 | Yes |
| UMA-4 | *U. macrophylla*_10 | KF881173 | Yes |
| UMA-6 | *U. macrophylla*_12 | KF881174 | Yes |
| UMA-8 | *U. macrophylla*_14 | KF881175 | Yes |
| PS1003MT01 | *U. sessilifructus*_01 | KM057036 | Yes |
| PS1003MT02 | *U. sessilifructus*_02 | KM057037 | Yes |
| USE-2 | *U. sessilifructus*_06 | KF881160 | Yes |
| USE-3 | *U. sessilifructus*_07 | KF881161 | Yes |
| USE-4 | *U. sessilifructus*_08 | KF881162 | Yes |
| PS1004MT01 | *U. hirsuta*_01 | KM057038 | Yes |
| PS1004MT02 | *U. hirsuta*_02 | KM057039 | Yes |
| PS1004MT03 | *U. hirsuta*_03 | KM057040 | Yes |
| PS1005MT01 | *U. lancifolia*_01 | KM057041 | Yes |
| ULA-1 | *U. lancifolia*_03 | KF881176 | Yes |
| PS1006MT01 | *U. homomalla*_01 | KM057042 | Yes |
| UHO-1 | *U. homomalla*_03 | KF881163 | Yes |
| UHO-2 | *U. homomalla*_04 | KF881164 | Yes |
| UHO-3 | *U. homomalla*_05 | KF881165 | Yes |
| UHO-4 | *U. homomalla*_06 | KF881166 | Yes |
| UHO-5 | *U. homomalla*_07 | KF881167 | Yes |
| UHO-6 | *U. homomalla*_08 | KF881168 | Yes |
| UHO-7 | *U. homomalla*_09 | KF881169 | Yes |
| PS1039MT01 | *U. sinensis*_01 | GQ435236 | Yes |
| USI-2 | *U. sinensis*_03 | KF881183 | Yes |
| USI-4 | *U. sinensis*_05 | KF881184 | Yes |
| UYU-1 | *U. yunnanensis*_01 | KF881191 | Yes |
| UYU-4 | *U. yunnanensis*_04 | KF881193 | Yes |
| UYU-5 | *U. yunnanensis*_05 | KF881194 | Yes |
| USC-1 | *U. scandens*_02 | KF881185 | Yes |
| USC-2 | *U. scandens*_03 | KF881186 | Yes |
| USC-3 | *U. scandens*_04 | KF881187 | Yes |
| USC-4 | *U. scandens*_05 | KF881188 | Yes |
| USC-6 | *U. scandens*_07 | KF881189 | Yes |
| USC-7 | *U. scandens*_08 | KF881190 | Yes |
| ULAE-1 | *U. laevigata*_02 | KF881179 | Yes |
| ULAE-4 | *U. laevigata*_05 | KF881180 | Yes |
| ULAE-5 | *U. laevigata*_06 | KF881181 | Yes |
| ULAE-7 | *U. laevigata*_08 | KF881182 | Yes |
| Total No. of sequences |  | 49 |  |

KF881172 inversion


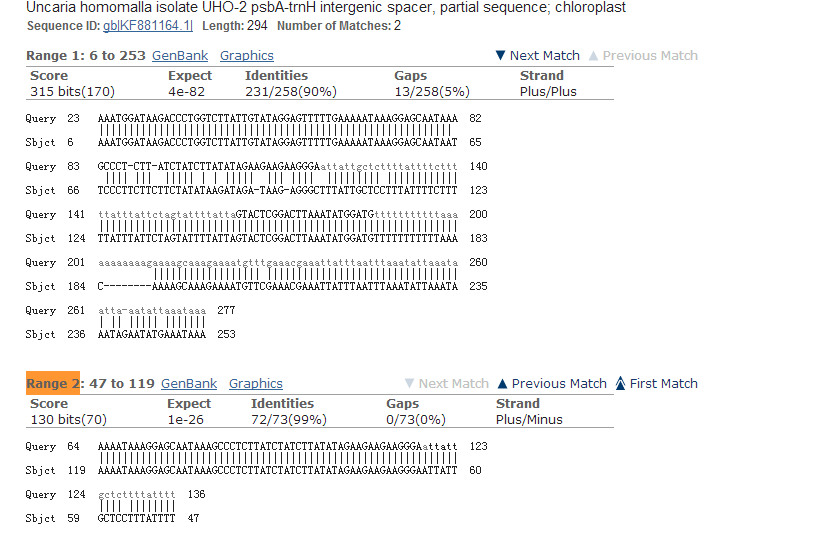

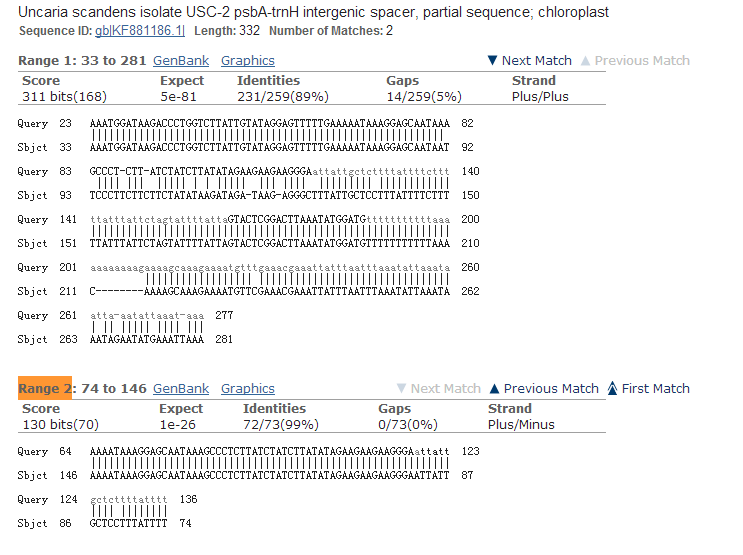

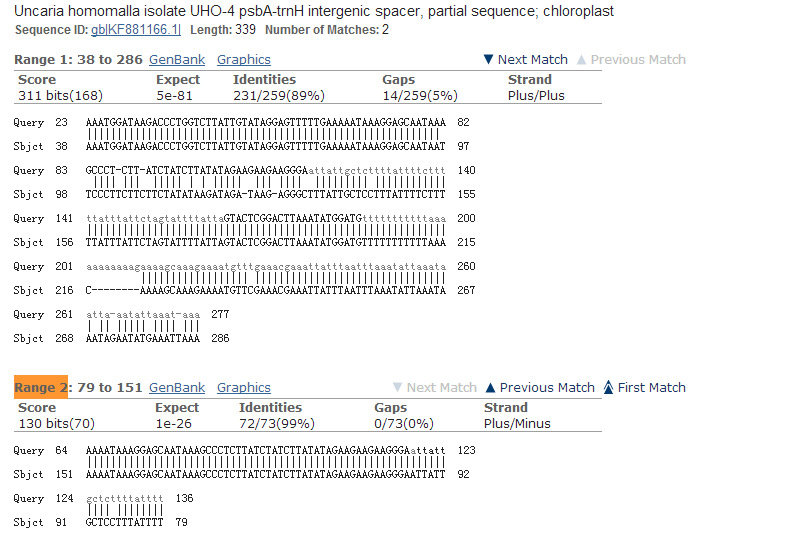

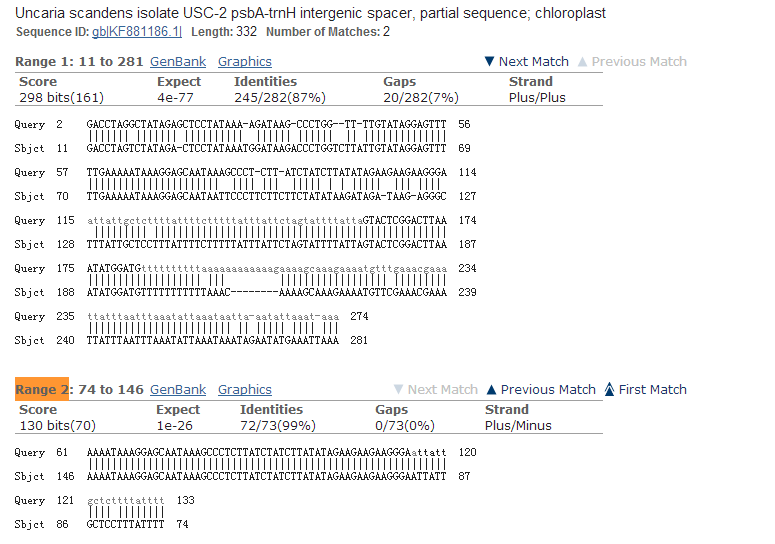


KF881174


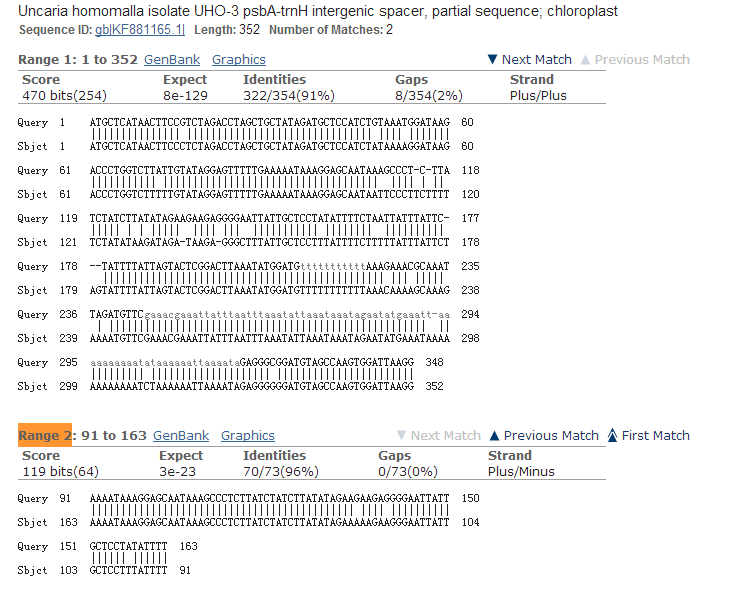


KF881160


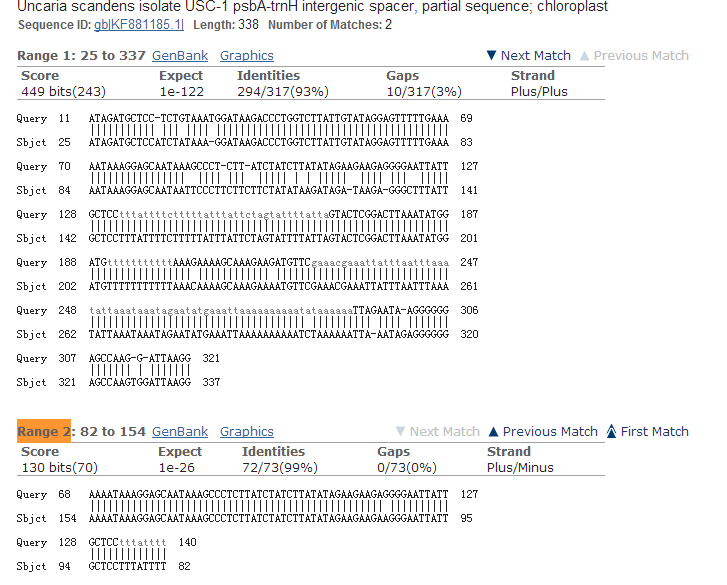


KF881161


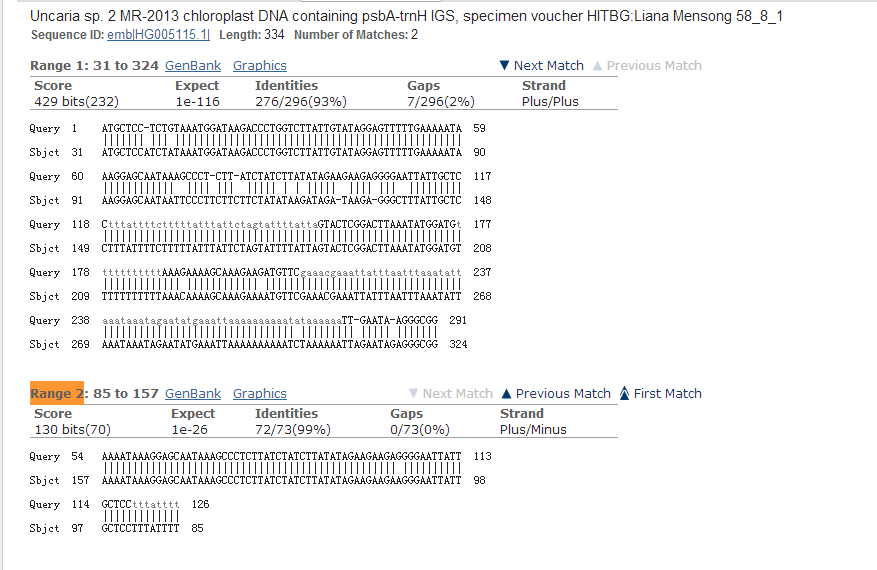


KF881162


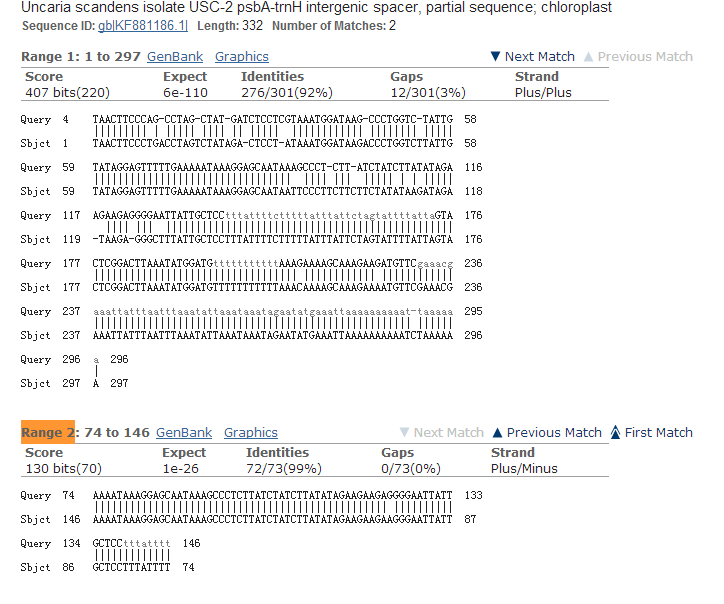

Supplement: Supplementary file 2 — 10.1186/s13020-015-0072-7 The accessions containing inversion sequence in psbA-trnH of Uncaria. [file 13020_2015_72_MOESM2_ESM.docx]
